# Point-of-care test of blood *Plasmodium* RNA within a Pasteur pipette using a novel isothermal amplification without nucleic acid purification

**DOI:** 10.1186/s40249-024-01255-8

**Published:** 2024-10-31

**Authors:** Lyu Xie, Jiyu Xu, Lihua Fan, Xiaodong Sun, Zhi Zheng

**Affiliations:** 1 Institute of Basic Medical Sciences, Chinese Academy of Medical Sciences, School of Basic Medicine, Peking Union Medical College, No. 5, Dongdansantiao, Dongcheng District, Beijing, 10005 China; 2 Yunnan Institute of Parasitic Diseases & Yunnan Provincial Centre of Malaria Research, Pu’er, China

**Keywords:** Point-of-care test, Nucleic acid extraction-free, Pasteur pipette, Isothermal probe amplification, Malaria

## Abstract

**Background:**

Resource-limited regions face a greater burden of infectious diseases due to limited access to molecular tests, complicating timely diagnosis and management. Current molecular point-of-care tests (POCTs) either come with high costs or lack adequate sensitivity and specificity. To facilitate better prevention and control of infectious diseases in underserved areas, we seek to address the need for molecular POCTs that better align with the World Health Organization (WHO)’s ASSURED criteria—Affordable, Sensitive, Specific, User-friendly, Rapid and robust, Equipment-free, and Deliverable to end users.

**Methods:**

A novel molecular POCT, Pasteur Pipette-assisted isothermal probe amplification (pp-IPA), was developed for malaria detection. Without any microfluidics, this method captures *Plasmodium* 18S rRNA in a modified Pasteur pipette using tailed genus-specific probes. After washing, the bound tailed probes are ligated to form a template for subsequent novel isothermal probe amplification using a pair of generic primers, bypassing nucleic acid extraction and reverse transcription. The method was assessed using cultured *Plasmodium* and compared with real-time quantitative reverse transcription PCR (RT-qPCR) or reverse transcription loop-mediated isothermal amplification (RT-LAMP) in clinical blood samples.

**Results:**

The entire assay is completed in 60–80 min with minimal hands-on time, using only a Pasteur pipette and a water bath. The pp-IPA’s analytical sensitivity is 1.28 × 10^–4^ parasites/μl, with 100% specificity against various blood-borne pathogens causing malaria-like symptoms. Additionally, pp-IPA needs only liquid-transfer skill for operation and the cost is around USD 0.25 per test, making it at least 300 times lower than mainstream POCT platforms.

**Conclusions:**

Designed to improve the accessibility of molecular detection in resource-limited settings, pp-IPA’s simplicity, affordability, high sensitivity/specificity, and minimal equipment requirements make it a promising point-of-care pathogen identification tool in resource-constrained regions.

**Graphical Abstract:**

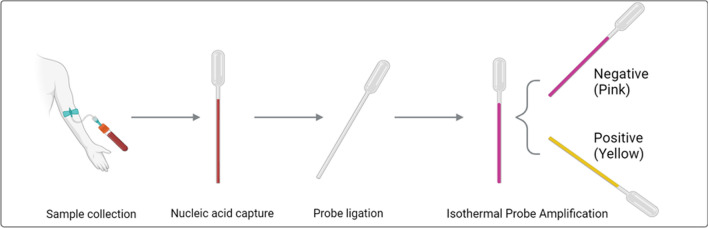

**Supplementary Information:**

The online version contains supplementary material available at 10.1186/s40249-024-01255-8.

## Background

The World Health Organization (WHO) estimates that infections contribute to almost 30% of the global disease burden. However, this burden is disproportionately distributed, with developed countries experiencing 4.6% and low-income states facing 58.1% [1]. Limited access to molecular tests is identified as a significant factor contributing to this regional disparity [2], and this limitation stems from the requirements of expensive equipment, complex procedures, and well-trained personnel for existing gold-standard assays [2]. To address this challenge, molecular point-of-care tests (POCTs) have emerged as a promising solution, presenting opportunities to overcome traditional diagnostic barriers and facilitate quicker, simpler and more cost-effective pathogen identification in areas with limited access to advanced medical infrastructure [3], thereby reducing the disease burden in resource-limited regions.

Over the last decade, microfluidics as one of the most promising molecular POCT platforms has garnered significant attention for miniaturizing operation procedures into chips [4], with specific applications envisioned in four scenarios, namely (1) clinics with accessories, (2) fields lacking personnel and equipment, (3) low-acuity clinics with budget constraints, and (4) fields without budgets [3]. Nevertheless, cutting-edge microfluidic-based molecular POCT platforms, such as GeneXpert from Cepheid, FilmArray from BioFire and xTAG from Luminex, predominantly find applications in the first two scenarios only. This is attributed to the fact that for a highly automated workflow, these platforms rely entirely on a specially designed and often expensive device/instrument for assay execution and signal detection, leading to considerable assay set-up costs. The use of a disposable microfluidic chip with complex design for assay integration results in additional significant costs. For example, the per-assay consumable cost is USD 78/test for GeneXpert [5], USD 180/test for FilmArray and USD 72/test for xTAG [6], limiting their widespread adoption in resource-limited settings.

To promote accessibility of molecular POCTs in resource-limited regions, the WHO introduced the "ASSURED" criteria for developing POCTs tailored to such settings, defining that the ideal molecular POCTs should be Affordable, Sensitive, Specific, User-friendly, Rapid and robust, Equipment-free, and Deliverable to end users [7]. Towards this goal, paper-based POCT platforms, alongside microfluidics, were developed [8], although few went beyond laboratory development to become commercially available [9]. Recently, the application of isothermal amplification techniques, particularly loop-mediated isothermal amplification (LAMP), in POCTs, has garnered increasing attention due to its intrinsic characteristics [10–12]. For instance, its thermal cycling-free advantage renders it independent of expensive instruments, while its high amplification speed enables shorter sample-to-answer times [13]. Additionally, its resistance to inhibitors [14] opens up the possibility of nucleic acid extraction-free methods. These characteristics collectively make them compatible for hand-held equipment in field settings. Building on these advantages, extraction-free LAMP POCTs were developed; however, they demonstrated a sensitivity of less than 50% [15] and frequently resulted in false negatives [16]. Consequently, nucleic acid extraction is typically integrated into most POCTs to guarantee analytical performance, compromising portability and affordability. To better meet the ASSURED criteria, paper-origami-based POCTs were developed, where nucleic acid was purified and directly amplified on the paper [17]. While cost-effectiveness and portability were easily achieved, the sensitivity was hindered by the rough nucleic acid extraction step, and there was a risk of cross-contamination during nucleic acid and amplicon transfer due to the open-system design [18, 19]. Recently, efforts have been made to prevent cross-contamination by creating a closed system using Pasteur pipettes; however, nucleic acid purification remains necessary [20, 21].

Given the lack of sensitive and affordable molecular POCTs in resource-limiting countries, we aim to develop Pasteur pipette-assisted isothermal probe amplification (pp-IPA), a novel molecular POCT that better meets the ASSURED criteria, using malaria detection as an example. Without the expensive microfluidics but utilizing a Pasteur pipette as a multifunctional tool for sample handling, target capture, and isothermal amplification, pp-IPA eliminates the need for nucleic acid extraction and specialized equipment, simplifying the diagnostic process and reducing costs. The only skill requirement for the operator is the ability to transfer liquid properly using a Pasteur pipette. In clinical sample evaluation, pp-IPA results agreed well with conventional molecular methods, proving its sensitivity, specificity, and flexibility as a molecular POCT solution for resource-limited areas, with the potential to extend beyond malaria identification.

## Methods

### Materials and reagents

Materials including 3× lysis buffer, 5× ligation mixture, 96-well capture plate, and modified Pasteur pipette were obtained from Diacurate (Beijing, China). Colorimetric IPA Master Mix (HMD5204) and RT-LAMP master mix (HMD5203) were purchased from Hzymes Biotechnology (Wuhan, China), while the real-time PCR premix (RR390) was procured from TAKARA Biotechnology (Dalian, China). The RNA purification kit (DP433) and proteinase K (RT403) were purchased from Tiangen Biotech (Beijing, China). The one-step RT-qPCR mix (Q222) was sourced from Vazyme (Jiangsu, China). Genus specific probes and primers used for this study (Table S1 and Fig. S1) were synthesized by Sangon (Shanghai, China). RT-qPCR and RT-LAMP were conducted on a LightCycler Real-Time PCR System (Roche, Basel, Switzerland) and a water bath (Qiwei Instrument Co., Ltd., Hangzhou, China) was applied for pp-IPA.

Cultured *Plasmodium falciparum* 3D7 strain was donated by Professor Wang H. of Chinese Academy of Medical Sciences and the density was determined using droplet digital PCR [23]. Dengue virus (DENV, including serotypes 1, 2, 3, and 4), Zika virus (ZIKV), chikungunya virus (CHIKV), and Japanese encephalitis virus (JEV) were graciously provided by the Yunnan Institute of Parasitic Diseases and quantified using RT-qPCR [24].

### RT-qPCR for conventional malaria determination

Extracted RNA was amplified in 20 μl RT-qPCR reaction mixture containing 400 nmol/L forward primer, 400 nmol/L reverse primer, 200 nmol/L hydrolysis probe [25], 5 μl RNA template and 10 μl 2× one-step RT-qPCR mix using the following thermal procedure: 50 °C for 5 min, 95 °C for 20 s, followed by 40 cycles of 95 °C for 10 s, 60 °C for 30 s.

### RT-LAMP malaria determination

Five microliter of extracted RNA from an appropriate amount of *Plasmodium* sample was amplified in a 25 µl LAMP reaction mixture containing 1.6 µmol/L FIP and BIP, 0.8 µmol/L LPF and LPB, 0.2 µmol/L F3 and B3 primers and 6.25 μl 4× RT-LAMP master mix, and incubated at 65 °C for 60 min [26].

### Procedures for pp-IPA

In a sampling tube, ten microliters of thawed whole blood or cultured *P. falciparum* 3D7 strain were lysed into a final volume of 50 μl lysate containing 1× lysis buffer, 1 nmol/L Ligation Probes (LPs), 1 nmol/L Capture Probes (CPs) and 1 μg/μl Proteinase K, using the Pasteur pipette to pipette up and down several times for thorough mixing. The lysate was then pipetted into the Pasteur pipette. The Pasteur pipette was sealed using a handheld mini hair-dress sealer and incubated in a 55 ℃ water bath for 30 min to facilitate the capture of 18S rRNA. After capture, the seal was cut off with a scissor and the content was expelled. The Pasteur pipette then underwent two washes using 150 μl of washing buffer and 0.1× SSC, respectively. Following the washes, fifty microliters of the ligation mixture were drawn into the pipette and incubated for 10 min at room temperature. After the ligation mixture was expelled, twenty-five microliters of the pink isothermal amplification mix, containing 12.5 μl of 2× Colorimetric IPA Master Mix, 800 nmol/L forward primer, and 800 nmol/L reverse primer, was drawn into the Pasteur pipette. The pipette was again sealed with a handheld mini hair-dressing sealer and placed in a 65 ℃-water bath to initiate IPA. For visual assessment, a color change from pink to yellow in the positive reaction mix could be observed at a defined time by the naked eye, while negative samples showed no color change.

### Sensitivity and specificity of pp-IPA

The sensitivity (limit of detection, LoD) of pp-IPA was evaluated using fivefold serial dilutions of *P. falciparum* 3D7 standard lysates, ranging from 2 to 0.0001 parasites/μl. Specificity was assessed against cultured dengue virus, Zika virus, chikungunya virus, and Japanese encephalitis virus. All tests were conducted according to the above pp-IPA procedures, with samples quantified by RT-qPCR in parallel to pp-IPA.

### Clinical sample test

Whole blood samples were gathered near Laiza City, Myanmar during the malaria transmission season in 2017 and malaria screening was carried out using both microscopy and qPCR [22]. Negative controls consisted of blood samples from individuals with no travel history to malaria-endemic areas and in good health. Both positive and negative blood samples were stored at −80 ℃ within 2 h after collection, and thawed without special requirements or pretreatment before the test. All samples were tested using pp-IPA following the above procedures.

### Statistical analysis

The Cq values or threshold times from RT-qPCR and RT-LAMP/pp-IPA were reported as mean ± standard deviation (SD). Sensitivity, specificity, and agreement between pp-IPA and RT-qPCR were calculated using SPSS 25.0 (IBM, New York, USA). The kappa coefficient, with a 95% confidence interval, was used to express agreement beyond chance.

## Results

### Development of pp-IPA for malaria detection

**Fig. 1:**
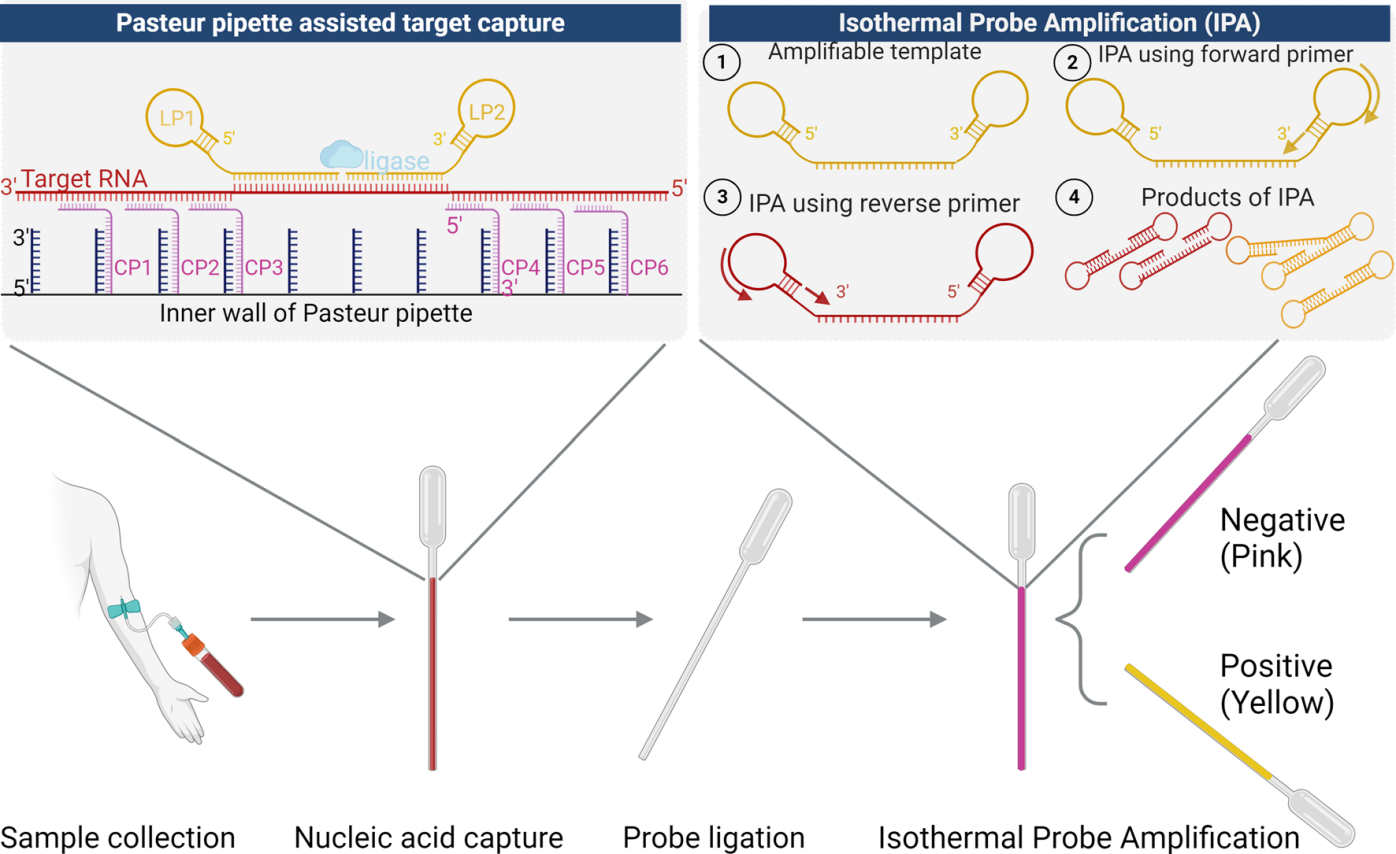
The schematic overview of pp-IPA. The Pasteur Pipette-assisted isothermal probe amplification (pp-IPA) involves three key steps: lysis and capture, ligation of probes, and amplification of templates (*bottom*). In the initial lysis and capture step (*top left*), RNAs were released from the cell after lysis and hybridized to the oligonucleotide hybridization probes (CP and LP) targeting highly conserved region of 18S rRNA of *Plasmodium* species. Each CP contains a target-specific portion and a universal 3ʹ -tail sequence for binding to the oligos on the inner wall of the Pasteur pipette, while the two LPs, which bind to two contiguous sequences of the target with their target-specific portions, have universal sequences forming stem-loop structure in their respective 5ʹ and 3ʹ portion. After washing off unbound probes, LPs (LP1 and LP2) are ligated to form a complete dumbbell-like template, a process only possible in the presence of captured target RNA. The colorimetric IPA process follows an isothermal amplification using Bst polymerase and one pair of generic primers binding to the predesigned generic loop region of the LP probes (*top right*). Following IPA, the positive sample reaction mix undergoes a change from pink to yellow, which aids in the visual identification by naked-eye observation, while negative samples maintain a pink coloration (*bottom*)

The schematic overview of the pp-IPA assay was presented in Fig. 1. In this approach, genus-specific, tailed probes are designed based on conserved regions of the 18S rRNA genes from the five human-infecting *Plasmodium* species (Fig. S1 and Table S1). They are used after cell lysis to capture 18S rRNA of *Plasmodium* spp. onto the inner wall of oligo-conjugated Pasteur pipettes through sandwich hybridization. After washing, the bound tailed probes are ligated to form complete dumbbell-shaped templates for subsequent novel isothermal probe amplification using a pair of primers, bypassing nucleic acid extraction and reverse transcription (Fig. 1).

**Fig. 2:**
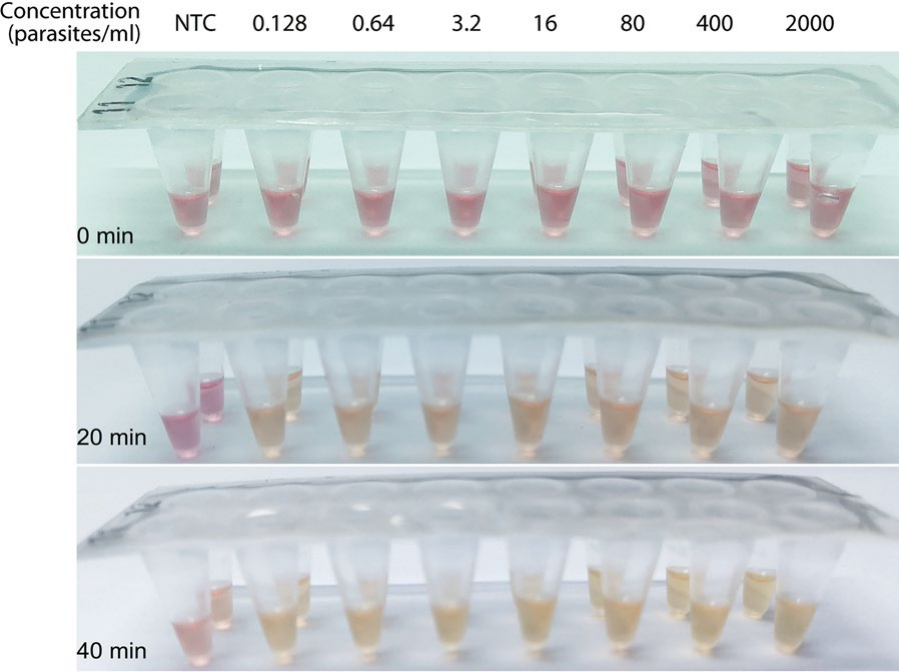
The colorimetric IPA assay for malaria detection utilizing oligo-conjugated 96-well PCR plates. A fivefold serial-dilution series was made from the lysate of a cultured 3D7 standard strain. The parasite concentrations ranged from 0.000128 to 2 parasites/μl and were marked on top. The results were observed at 0, 20, and 40 min, respectively. Each sample was run in duplicates. *IPA* isothermal probe amplification, *ND* not detected

The method’s feasibility was initially assessed using an oligo-conjugated 96-well PCR plate to test cultured standard *P. falciparum* 3D7 strain lysate (Fig. 2). The parasite concentrations ranged from 0.000128 to 2 parasites/μl. Following the amplification, all parasite concentrations could be clearly distinguished by color from the negative control within 20 min. It is noteworthy that the intensity of the color change was independent of parasite concentration.

### Sensitivity and specificity of pp-IPA

**Fig. 3:**
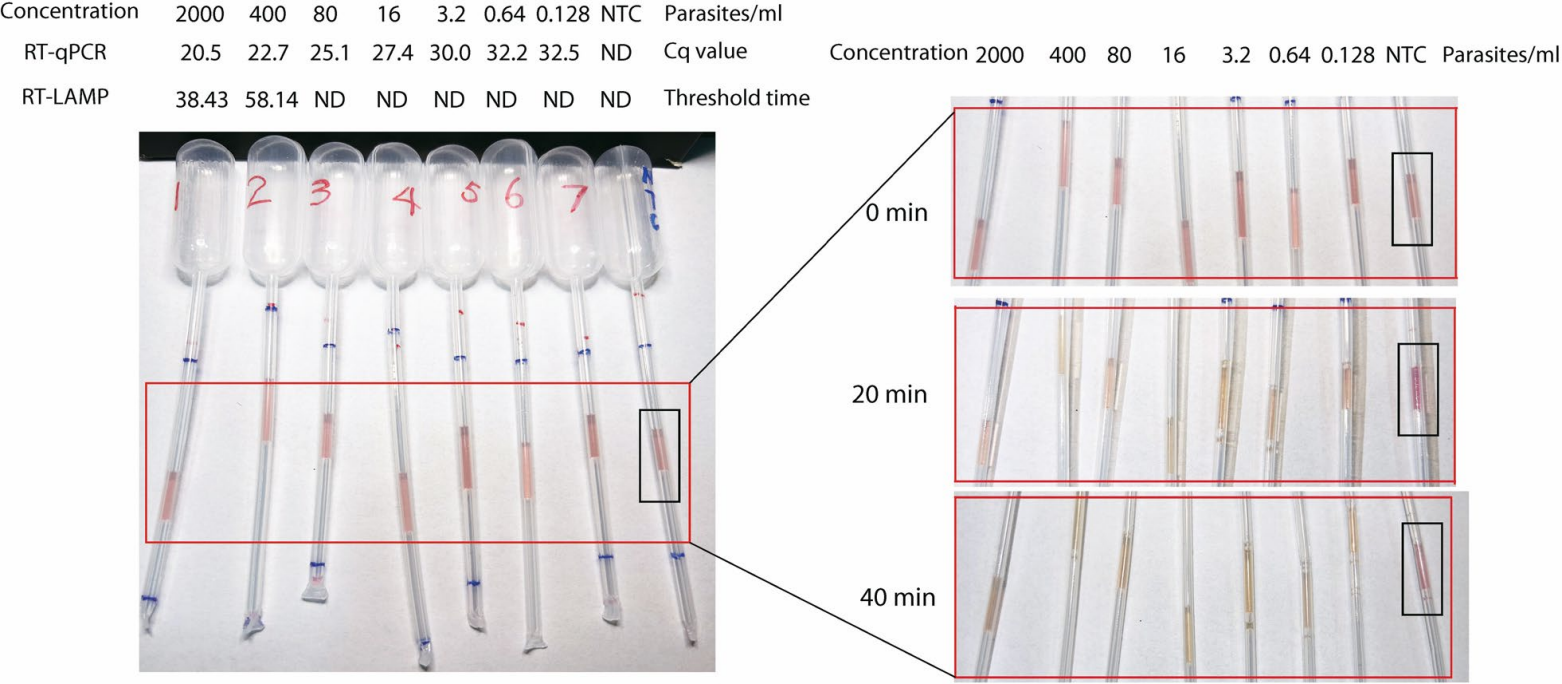
The establishment of colorimetric pp-IPA assay for malaria detection. A fivefold dilution series made from lysate of a cultured 3D7 standard strain was assayed using colorimetric pp-IPA. The results were observed at 0, 20, and 40 min, respectively. The *left image* shows an overview of pp-IPA amplification, with the *red box* indicating the amplification result observation area and the *black rectangle* indicating the no template control (NTC). The *right image* is an enlarged view of the result observation area. The concentrations ranged from 0.000128 to 2 parasites/μl and were marked above each sample. The associated RT-qPCR and RT-LAMP results for each sample were listed as well for comparison. Each sample was run in triplicates and one representative pipette was shown. *pp-IPA* Pasteur pipette-assisted isothermal probe amplification, *ND* not detected

After confirming feasibility, the 96-well PCR plate was replaced with oligo-conjugated Pasteur pipettes while maintaining all other conditions unchanged for the IPA method. Results showed that across all concentrations ranging from 2 to 1.28 × 10^–4^ parasites/μl, a notable pink-to-yellow color change, clearly distinguishable from the negative control, was observed, while the negative control remained distinctly pink after 40 min of amplification (Fig. 3). Therefore, the analytical sensitivity of the pp-IPA was set to be at least 1.28 × 10^–4^ parasites/μl. The positive samples changed color between 20 ± 5 and 40 ± 3 min, with the intensity of the color change being independent of parasite concentration (Fig. 3).

**Fig. 4:**
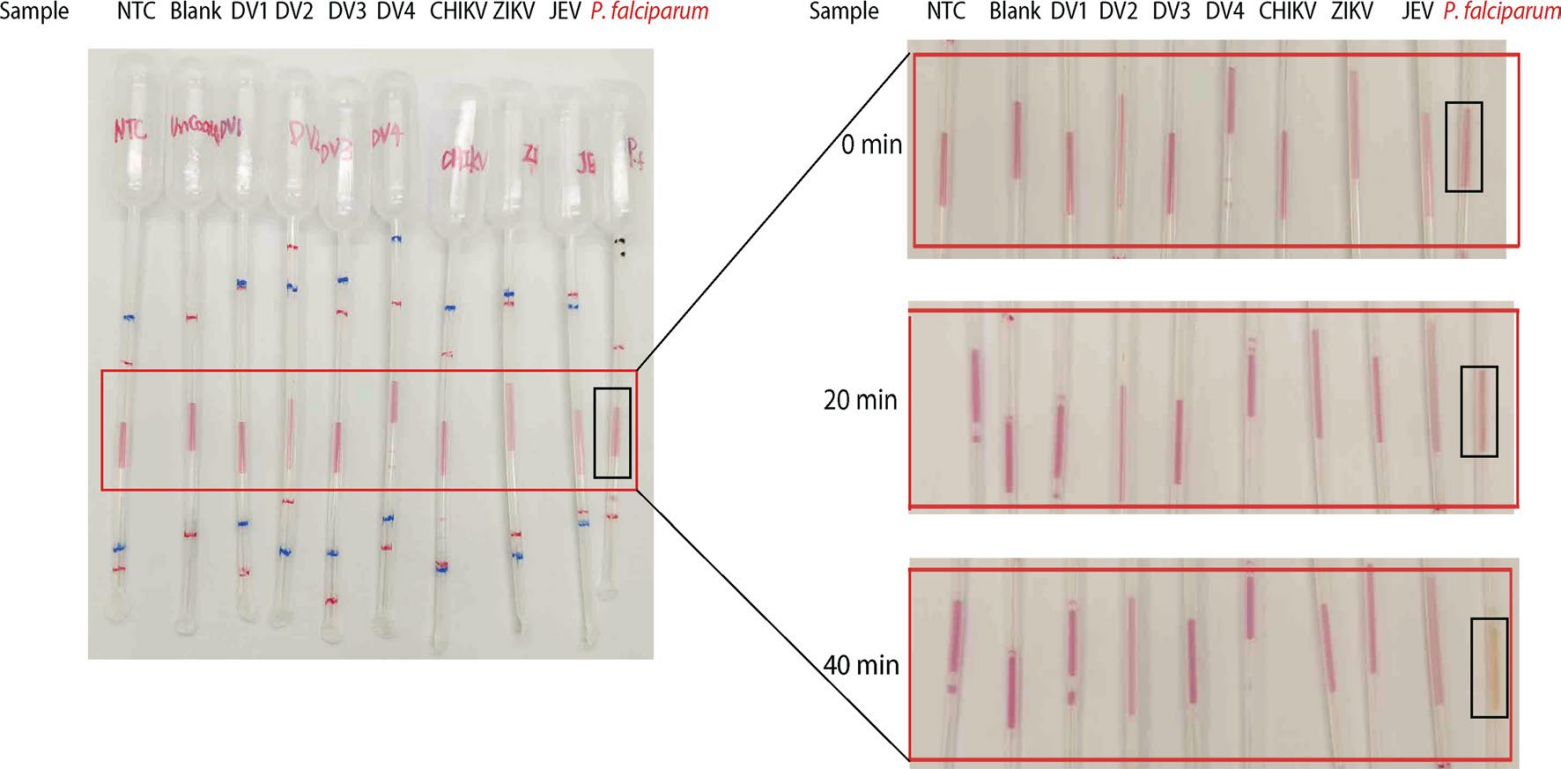
Specificity of pp-IPA. The specificity of pp-IPA was examined by amplifying DENV1, DENV2, DENV3, DENV4, ZIKV, CHIKV, and JEV using malaria CPs, LPs, and primers. Blank Pasteur pipette (no oligo conjugated) and NTC were set as negative controls. The *left image* shows an overview of pp-IPA, with the *red box* indicating the amplification result observation area and the black rectangle indicating the positive control for *P. falciparum.* The *right image* is an enlarged view of the amplification result observation area. Each sample was run in triplicates and one representative pipette was shown. The associated Cq values of DENV1, DENV2, DENV3, DENV4, ZIKV, CHIKV, JEV and *P. falciparum* from viral- and *Plasmodium*-specific RT-qPCR were 18.6 (± 0.4), 23.8 (± 0.2), 15.9 (± 0.5), 21.6 (± 0.4), 23.0 (± 0.7), 22.9 (± 0.1), 19.1 (± 0.6), 19.3 (± 0.2), respectively. *pp-IPA* Pasteur pipette-assisted isothermal probe amplification, *DENV1* dengue virus serotype 1, *DENV2* dengue virus serotype 2, *DENV3* dengue virus serotype 3, *DENV4* dengue virus serotype 4, *ZIKV* Zika virus, *CHIKV* chikungunya virus, *JEV* Japanese encephalitis virus, *NTC* no template control

To assess assay specificity, cultured dengue virus, Japanese encephalitis virus, Zika virus, and chikungunya virus at high concentrations were tested together with a *P. falciparum* positive control. No amplification occurred for any of the viruses, while a clear color change was observed for the *P. falciparum* positive control (Fig. 4), demonstrating a 100% specificity of pp-IPA in malaria detection.

### Clinical sample test

**Fig. 5:**
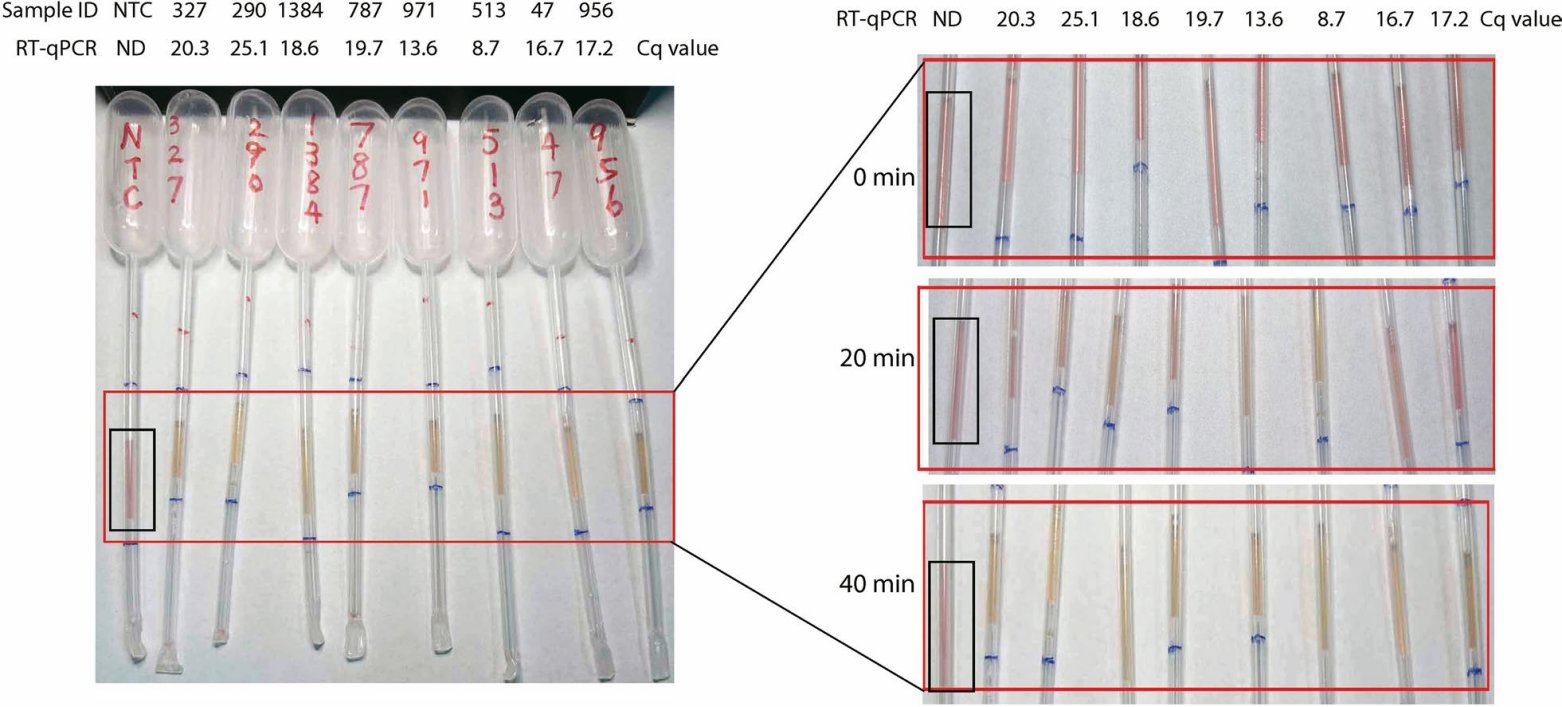
Clinical samples validation by pp-IPA. Eight clinical samples were amplified using pp-IPA, the red rectangle indicates the area containing the IPA mix for result readout, and color change of reaction mix was observed at 0, 20 and 40 min three time points. The Cq values of each sample, as determined by RT-qPCR were recorded below the sample ID numbers. Each sample was run in triplicates and one representative pipette was shown. *pp-IPA* Pasteur pipette-assisted isothermal probe amplification, *ND* not detected

Sixty-two suspected malaria samples from a malaria-endemic region in Myanmar, along with two healthy blood samples, were chosen for the validation of pp-IPA. In the assays, 47 samples exhibited a clear color change from pink to yellow, while 15 samples and the healthy control retained their pink colors (Fig. 5 and Fig. S2). To quantitatively verify the accuracy of pp-IPA, RT-qPCR was employed as the gold standard for assessing the sensitivity and specificity of pp-IPA in detecting clinical samples. The results of pp-IPA showed perfect agreement with RT-qPCR (Fig. 5 and Fig. S2), with a kappa value of 1.0. This high level of concordance underscores the reliability and accuracy of pp-IPA in the detection of clinical malaria samples.

## Discussion

Although several molecular POCTs like Genexpert and Filmarray have found success in certain scenarios, they remain costly and dependent on specialized equipment due to the requirement of nucleic acid extraction and thermal cycling steps. Consequently, they fail to meet the ASSURED criteria for use in resource-limited settings [3]. While paper-based LAMP POCTs showed better potential in this regard, they frequently suffer from low sensitivity and false positives [15] due to crude nucleic acid extraction techniques [18, 19, 27] and the intrinsic limitations of LAMP [16], thereby impeding accessibility in resource-poor settings. Here, pp-IPA is specifically designed to overcome the above challenges for malaria diagnosis.

Unlike aforementioned POCT platforms, pp-IPA achieves cost-effectiveness using an extraction-free approach based on sandwich hybridization and target-dependent probe ligation (Fig. 1). It completes the process using only a Pasteur pipette for all liquid transfers as well as for amplification and result-readout, minimizing instrument requirement. The selection of Pasteur pipettes as the material for developing an ASSURED molecular POCT was driven by several factors. (1) Pasteur pipettes have a well-established history in analytical chemistry as droppers and are readily available worldwide [28]. They are low-cost, stable, lightweight, easy to store and transport. (2) Their transparency renders them ideal for colorimetric visualization. (3) The small diameter of the Pasteur pipette stem minimizes the consumption of reagents while still rendering the visual observation of color, reducing the cost per assay. (4) The thin-walled Pasteur pipettes can be easily heat-sealed to create a closed system for isothermal amplification, minimizing potential cross contamination of amplification. In our work, we primarily utilized polypropylene Pasteur pipettes with a diameter of 1 mm due to their excellent heat tolerance and high specific surface area [29].

Besides cost, pp-IPA’s targeted-capture design allows for excellent analytical performance: First, the assay is designed to detect the much abundant RNA targets, rendering a significantly higher sensitivity than other POCT techniques which detect DNAs. Additionally, the capturing probes (CPs) were designed to hybridize onto multiple sections of the RNA molecule (Fig. S1), leading to higher capture efficiency and sensitivity. Second, the complete sandwich hybridization formed by multiple probes and the target sequence (Fig. 1) is the prerequisite for subsequent ligation and amplification. Any RNA sequences that bind nonspecifically to individual probes either remain uncaptured or are unable to be amplified. This greatly increases the assay specificity. Third, background noise from nonspecific amplification was reduced by washing off non-target nucleic acid [16], and by the requirement that only ligated adjacently-bound probes can be amplified. Fourth, pp-IPA exclusively amplifies the ligated probes rather than pathogen RNA. This amplifiable template is not formed until after ligation, significantly reducing the potential for cross-contamination. Last, the RNA target and the ligated probe were anchored by sandwich hybridization to the Pasteur pipette inner surface rather than in the solution, rendering pp-IPA less susceptible to template-containing aerosol generation, reducing chances of contamination. As a result, pp-IPA demonstrates an excellent sensitivity as low as 1.28 × 10^–4^ parasites/μl, surpassing existing malaria POCTs by three to four orders of magnitude (Table 1), and exhibits no nonspecific amplification for DENV, JEV, ZIKV, CHIKV, healthy blood, and NTC (Fig. 4).

**Table 1:** Performance comparison of pp-IPA with other existing POCTs for malaria detection

Technique	Number of primers	NA extraction	LoD (Parasites/μl)	Visualization	Open/closed system for amplification/visualization^1^	Equipment	Non-reagent consumable
pp-IPA	2	No	0.000128	Colorimetric	Closed	Water bath	Pasteur pipette
RPA[33]	2	No	Not stated	Lateral flow assay	Open	Heating block	Pipette, tips, strip
LAMP[34]	4	No	3	Lateral flow assay	Open	Water bath	Streptavidin-coated beads, microfluidic, pipette, tips
LAMP[35]	4–6	Extraction kit	1	Fluorescence	Closed	Portable LAMP device	DNA extraction kit, pipette, tips
LAMP[17]	6	Glass fiber	50	Fluorescence	Open	Handheld UV lamp	Paper microfluidic, acetate film, pipette, tips
LAMP	4	Magnetic beads	0.5–50	LFA[36]/Fluorescence[37]/Turbidity[38]	Open[36]/Closed[37, 38]	Portable LAMP device	Microfluidic, acetate films, beads, strip, pipette, tips
RPA	2	Extraction kit	1–4	Lateral flow assay/Colorimetric	Open[39]/Closed[40]	Heating block	Pipette, tips, strip
CRISPR-SHERLOCK	2	Extraction kit	2	Lateral flow assay	Open[41]	Heating block	Extraction kit, Pipette, tips

1: A closed system is critical for avoiding cross contamination

*pp-IPA* Pasteur pipette-assisted isothermal probe amplification, *RPA* recombinase polymerase amplification, *LAMP* loop-mediated isothermal amplification, *SHERLOCK* specific high-sensitivity enzymatic reporter unlocking, *CRISPR* clustered regularly interspaced short palindromic repeats, *NA* nucleic acid, *LoD* limit of detection, *LFA* lateral flow assay

Furthermore, our novel isothermal amplification technology, IPA, also contributes to the improved analytical performance. It uses only one pair of optimized generic, target-unrelated primers for each target, instead of two or three pairs of target-specific primers as in LAMP amplification. This greatly simplifies primer design, while considerably minimizes the potential for false positives due to nonspecific priming arising from primer interactions, which is one of the most significant issues regarding isothermal amplification such as LAMP. Moreover, IPA can be initiated without “breathing” of double stranded DNA for primer-binding, which is an unfavorable requirement for initiating LAMP and LAMP-like amplifications such as cross priming amplification [30] and strand exchange amplification [31]. This results in higher amplification efficiency and speed. Throughout our testing, IPA identified targets as fast as 7 min and exhibited no amplification signal for the NTC up to 90 min (Fig. S3).

This study introduces pp-IPA for the identification of *Plasmodium* RNA, making a significant contribution to point-of-care molecular approaches for pathogen detection in resource-limited areas. The pp-IPA demonstrates better characteristics than other existing POCTs of malaria (Table 1), such as no RNA extraction, easier primer design, higher sensitivity and specificity, least consumption of non-reagent consumable, least likelihood of cross-contamination, and nearly instrument-free operation. The only instrument required is a water bath, and the total assay cost was less than USD 0.25 per test (primers and probes: USD 0.0004, Pasteur pipette: USD 0.0015, ligation reagent: USD 0.048, IPA reagent: USD 0.20).

There are still aspects that can be further improved, for example the potential combination of ligation and amplification into a single step, and the development of a complete instrument-free assay by replacing the water bath with the use of an inexpensive, self-heating disposable heat patch [32]. Furthermore, lyophilized IPA reagents, which are already available (HaiGene, Cat # A3810), can offer the advantage of transportation and storage at room temperature for over 6 months. These features will significantly enhance pp-IPA’s accessibility for malaria detection in resource-limited settings. As one of the POCTs most closely aligned with all the ASSURED criteria (Table 1), pp-IPA holds great potential for effectively addressing diagnostic challenges in regions with constrained resources.

## Conclusions

Overall, pp-IPA as a novel molecular POCT for malaria detection, can address the key limitations of current diagnostic methods in resource-limited settings. The test is low-cost, priced at approximately USD 0.25 per test, and easy to use, requiring only a water bath and basic pipetting skills. It is also highly reliable, with a limit of detection comparable to RT-qPCR at 0.000128 parasites/μl and demonstrating 100% specificity. These characteristics offer significant potential to expand molecular testing in underserved regions and advance efforts to combat malaria and other infectious diseases.

## Supplementary Information

Additional file 1.

## Data Availability

All data generated or analyzed during this study are included in this published article [and its supplementary information files].

## Abbreviations

POCT: Point-of-care test
WHO: World Health Organization
pp-IPA: Pasteur pipette-assisted isothermal probe amplification
LAMP: Loop-mediated isothermal amplification
rRNA: Ribosomal RNA
DENV: Dengue virus
ZIKV: Zika virus
CHIKV: Chikungunya virus
JEV: Japanese encephalitis virus
LP: Ligation probe
CP: Capture probe
RT-qPCR: Real-time reverse transcription PCR
SSC: Saline-sodium citrate buffer
NTC: No template control
RPA: Recombinase polymerase amplification

## References

[1] Burden of Disease. https://ourworldindata.org/burden-of-disease. Access 12 October 2024.

[2] Land KJ, Boeras DI, Chen XS, Ramsay AR, Peeling RW. REASSURED diagnostics to inform disease control strategies, strengthen health systems and improve patient outcomes. Nat Microbiol. 2019;4(1):46–54. 30546093 10.1038/s41564-018-0295-3 PMC7097043

[3] Gavina K, Franco LC, Khan H, Lavik JP, Relich RF. Molecular point-of-care devices for the diagnosis of infectious diseases in resource-limited settings–A review of the current landscape, technical challenges, and clinical impact. J Clin Virol. 2023;169: 105613. 37866094 10.1016/j.jcv.2023.105613

[4] Wang X, Hong XZ, Li YW, Li Y, Wang J, Chen P, et al. Microfluidics-based strategies for molecular diagnostics of infectious diseases. Mil Med Res. 2022;9(1):1–27. 35300739 10.1186/s40779-022-00374-3 PMC8930194

[5] Kaso AW, Hailu A. Costs and cost-effectiveness of Gene Xpert compared to smear microscopy for the diagnosis of pulmonary tuberculosis using real-world data from Arsi zone, Ethiopia. PLoS ONE. 2021;16(10): e0259056. 34695153 10.1371/journal.pone.0259056 PMC8544827

[6] DiDiodato G, Allen A, Bradbury N, Brown J, Cruise K, Jedrzejko C, et al. The efficacy of the BioFire FilmArray gastrointestinal panel to reduce hospital costs associated with contact isolation: a pragmatic randomized controlled trial. Cureus. 2022;14(8): e27931. 36120274 10.7759/cureus.27931 PMC9464456

[7] Mabey D, Peeling RW, Ustianowski A, Perkins MD. Diagnostics for the developing world. Nat Rev Microbiol. 2004;2(3):231–40. 15083158 10.1038/nrmicro841

[8] Martinez AW, Phillips ST, Whitesides GM, Carrilho E. Diagnostics for the developing world: microfluidic paper-based analytical devices. Anal Chem. 2010;82(1):3–10. 20000334 10.1021/ac9013989

[9] Hou Y, Lv CC, Guo YL, Ma XH, Liu W, Jin Y, et al. Recent advances and applications in paper-based devices for point-of-care testing. J Anal Test. 2022;6(3):247–73. 35039787 10.1007/s41664-021-00204-w PMC8755517

[10] Moehling TJ, Choi G, Dugan LC, Salit M, Meagher RJ. LAMP diagnostics at the point-of-care: emerging trends and perspectives for the developer community. Expert Rev Mol Diagn. 2021;21(1):43–61. 33474990 10.1080/14737159.2021.1873769

[11] Jirawannaporn S, Limothai U, Tachaboon S, Dinhuzen J, Kiatamornrak P, Chaisuriyong W, et al. Rapid and sensitive point-of-care detection of *Leptospira* by RPA-CRISPR/Cas12a targeting *lipL32*. PLoS Negl Trop Dis. 2022;16(1): e0010112. 34990457 10.1371/journal.pntd.0010112 PMC8769300

[12] Subsoontorn P, Lohitnavy M, Kongkaew C. The diagnostic accuracy of isothermal nucleic acid point-of-care tests for human coronaviruses: a systematic review and meta-analysis. Sci Rep. 2020;10(1):22349. 33339871 10.1038/s41598-020-79237-7 PMC7749114

[13] Atceken N, Munzer Alseed M, Dabbagh SR, Yetisen AK, Tasoglu S. Point-of-care diagnostic platforms for loop-mediated isothermal amplification. Adv Eng Mater. 2023;25(8):2201174.

[14] Wong YP, Othman S, Lau YL, Radu S, Chee HY. Loop-mediated isothermal amplification (LAMP): a versatile technique for detection of micro-organisms. J Appl Microbiol. 2018;124(3):626–43. 29165905 10.1111/jam.13647 PMC7167136

[15] Iacobucci G. Covid-19: Rapid test missed over 50% of positive cases in Manchester pilot. BMJ. 2020;371: m4323. 33158908 10.1136/bmj.m4323

[16] Kim SH, Lee SY, Kim U, Oh SW. Diverse methods of reducing and confirming false-positive results of loop-mediated isothermal amplification assays: a review. Anal Chim Acta. 2023:341693. 10.1016/j.aca.2023.341693 37858542

[17] Xu G, Nolder D, Reboud J, Oguike MC, van Schalkwyk DA, Sutherland CJ, et al. Paper-origami-based multiplexed malaria diagnostics from whole blood. Angew Chem Int Ed Engl. 2016;128(49):15476–9. 10.1002/anie.201606060 PMC5132111 27554333

[18] Rodriguez NM, Wong WS, Liu L, Dewar R, Klapperich CM. A fully integrated paperfluidic molecular diagnostic chip for the extraction, amplification, and detection of nucleic acids from clinical samples. Lab Chip. 2016;16(4):753–63. 26785636 10.1039/c5lc01392e PMC4747825

[19] Khaliliazar S, Toldrà A, Chondrogiannis G, Hamedi MM. Electroanalytical paper-based nucleic acid amplification biosensors with integrated thread electrodes. Anal Chem. 2021;93(42):14187–95. 34648274 10.1021/acs.analchem.1c02900 PMC8552215

[20] Kang J, Li Y, Zhao Y, Wang Y, Ma C, Shi C. Nucleic acid extraction without electrical equipment via magnetic nanoparticles in Pasteur pipettes for pathogen detection. Anal Biochem. 2021;635: 114445. 34740597 10.1016/j.ab.2021.114445 PMC8562038

[21] Liu S, Wei M, Liu R, Kuang S, Shi C, Ma C. Lab in a Pasteur pipette: low-cost, rapid and visual detection of *Bacillus cereu* using denaturation bubble-mediated strand exchange amplification. Anal Chim Acta. 2019;1080:162–9. 31409466 10.1016/j.aca.2019.07.011

[22] Cheng Z, Wang D, Tian X, Sun Y, Sun X, Xiao N, et al. Capture and ligation probe-PCR (CLIP-PCR) for molecular screening, with application to active malaria surveillance for elimination. Clin Chem. 2015;61(6):821–8. 25964304 10.1373/clinchem.2014.237115

[23] Koepfli C, Nguitragool W, Hofmann NE, Robinson LJ, Ome-Kaius M, Sattabongkot J, et al. Sensitive and accurate quantification of human malaria parasites using droplet digital PCR (ddPCR). Sci Rep. 2016;6(1):39183. 27982132 10.1038/srep39183 PMC5159915

[24] Xu Z, Peng Y, Yang M, Li X, Wang J, Zou R, et al. Simultaneous detection of Zika, chikungunya, dengue, yellow fever, West Nile, and Japanese encephalitis viruses by a two-tube multiplex real-time RT-PCR assay. J Med Virol. 2022;94(6):2528–36. 35146775 10.1002/jmv.27658

[25] Christensen P, Bozdech Z, Watthanaworawit W, Imwong M, Rénia L, Malleret B, et al. Reverse transcription PCR to detect low density malaria infections. Wellcome Open Res. 2021;6:39. 10.12688/wellcomeopenres.16564.1 PMC9086519 35592834

[26] Mohon AN, Getie S, Jahan N, Alam MS, Pillai DR. Ultrasensitive loop mediated isothermal amplification (US-LAMP) to detect malaria for elimination. Malar J. 2019;18:1–10. 31619258 10.1186/s12936-019-2979-4 PMC6796404

[27] Linnes JC, Fan A, Rodriguez NM, Lemieux B, Kong H, Klapperich CM. Paper-based molecular diagnostic for *Chlamydia trachomatis*. RSC Adv. 2014;4(80):42245–51. 25309740 10.1039/C4RA07911F PMC4188396

[28] Ridley J. Pipetting and use of glassware. In: Dave G, editor. Essentials of clinical laboratory science. New York: Delmar; 2010. p. 185–205.

[29] Eichhorn SJ, Sampson WW. Relationships between specific surface area and pore size in electrospun polymer fibre networks. J R Soc Interface. 2010;7(45):641–9. 19812071 10.1098/rsif.2009.0374 PMC2842785

[30] Xu G, Hu L, Zhong H, Wang H, Yusa S, Weiss TC, et al. Cross priming amplification: mechanism and optimization for isothermal DNA amplification. Sci Rep. 2012;2(1):246. 22355758 10.1038/srep00246 PMC3271364

[31] Shi C, Shang F, Zhou M, Zhang P, Wang Y, Ma C. Triggered isothermal PCR by denaturation bubble-mediated strand exchange amplification. Chem Commun (Camb). 2016;52(77):11551–4. 27602549 10.1039/c6cc05906f

[32] Zhang Y, Zhang L, Sun J, Liu Y, Ma X, Cui S, et al. Point-of-care multiplexed assays of nucleic acids using microcapillary-based loop-mediated isothermal amplification. Anal Chem. 2014;86(14):7057–62. 24937125 10.1021/ac5014332

[33] Lalremruata A, Nguyen TT, McCall MBB, Mombo-Ngoma G, Agnandji ST, Adegnika AA, et al. Recombinase polymerase amplification and lateral flow assay for ultrasensitive detection of low-density *Plasmodium falciparum* infection from controlled human malaria infection studies and naturally acquired infections. J Clin Microbiol. 2020;58(5):10–1128. 10.1128/JCM.01879-19 PMC7180247 32102854

[34] Colbert AJ, Co K, Lima-Cooper G, Lee DH, Clayton KN, Wereley ST, et al. Towards the use of a smartphone imaging-based tool for point-of-care detection of asymptomatic low-density malaria parasitaemia. Malar J. 2021;20(1):1–13. 34563189 10.1186/s12936-021-03894-w PMC8466697

[35] Vincent JP, Komaki-Yasuda K, Iwagami M, Kawai S, Kano S. Combination of PURE-DNA extraction and LAMP-DNA amplification methods for accurate malaria diagnosis on dried blood spots. Malar J. 2018;17(1):1–7. 30348162 10.1186/s12936-018-2527-7 PMC6196555

[36] Reboud J, Xu G, Garrett A, Adriko M, Yang Z, Tukahebwa EM, et al. Paper-based microfluidics for DNA diagnostics of malaria in low resource underserved rural communities. Proc Natl Acad Sci U S A. 2019;116(11):4834–42. 30782834 10.1073/pnas.1812296116 PMC6421471

[37] Choi G, Prince T, Miao J, Cui L, Guan W. Sample-to-answer palm-sized nucleic acid testing device towards low-cost malaria mass screening. Biosens Bioelectron. 2018;115:83–90. 29803865 10.1016/j.bios.2018.05.019 PMC6459019

[38] De Koninck A-S, Cnops L, Hofmans M, Jacobs J, Van den Bossche D, Philippé J. Diagnostic performance of the loop-mediated isothermal amplification (LAMP) based illumigene® malaria assay in a non-endemic region. Malar J. 2017;16:1–9. 29041927 10.1186/s12936-017-2065-8 PMC5645927

[39] Lin H, Zhao S, Liu Y, Shao L, Ye Y, Jiang N, et al. Rapid visual detection of *Plasmodium* using recombinase-aided amplification with lateral flow dipstick assay. Front Cell Infect Microbiol. 2022;12: 922146. 35811679 10.3389/fcimb.2022.922146 PMC9263184

[40] Lai MY, Lau YL. Detection of *Plasmodium knowlesi* using recombinase polymerase amplification (RPA) combined with SYBR Green I. Acta Trop. 2020;208: 105511. 32422380 10.1016/j.actatropica.2020.105511

[41] Lee RA, Puig HD, Nguyen PQ, Angenent-Mari NM, Donghia NM, McGee JP, et al. Ultrasensitive CRISPR-based diagnostic for field-applicable detection of *Plasmodium* species in symptomatic and asymptomatic malaria. Proc Natl Acad Sci U S A. 2020;117(41):25722–31. 32958655 10.1073/pnas.2010196117 PMC7568265

