# Supplementary material for: Point-of-care test of blood Plasmodium RNA within a Pasteur pipette using a novel isothermal amplification without nucleic acid purification
Source: Infect Dis Poverty. 2024 Oct 31;13:80. doi: 10.1186/s40249-024-01255-8 (PMC11526708; doi:10.1186/s40249-024-01255-8)
Supplement: Supplementary file 1 — Additional file 1. [file 40249_2024_1255_MOESM1_ESM.docx]

Table S1 Primers and probes used for pp-IPA

| Name | Sequence (5'-3') |
| --- | --- |
| S4LP1^1^ | CCCTTAACTTTCGTTCTTGATTTTATCGTCGTGACTGTTTGTAATAGGACAGAGCCCCGCACTTTCAGTCACGACGAT |
| S4LP2 | CGACAGCAGAGGATTTGTTGTGTGGAAGTGTGAGCGGATTTTCCTCTGCTGTCGTTTTGGTATCTGATCGTCTTCACT |
| S5CP1^2^ | TTTTCGGCGGAGGAA |
| S5CP2 | TCACGATATATATTGATAAAGATTACCTAC |
| S4CP1 | TCTAAGAATTTCACCTCTGACATCTG |
| S4CP2 | GCAGTTGTTCGTCTCCAGAAAA |
| S4CP3 | TCGGCATAGTTTATGGTTAAGATTA |
| S3CP1 | TGTCTTAAACTAGTGAGTTTCCCC |
| S3CP2 | AGCTATTAATCTGTCAATCCTACTCT |
| S1CP1 | AGCTGATAGGTCAGAAACTCGAT |
| S1CP2 | GGCCAATACCCTAACATCAAA |
| F^3^ | ATCGTCGTGACTGAAAGTGCGGGGCTCTGTCCTATTAC |
| R^4^ | CGACAGCAGAGGATTTGTTGTGTGGAAGTGTGAGCGGA |

1: LP: ligation probe, 2: CP: target-specific capture probe, 3: F: forward primer, 4: R: reverse primer. S1 – S4 mean the four sections for capture


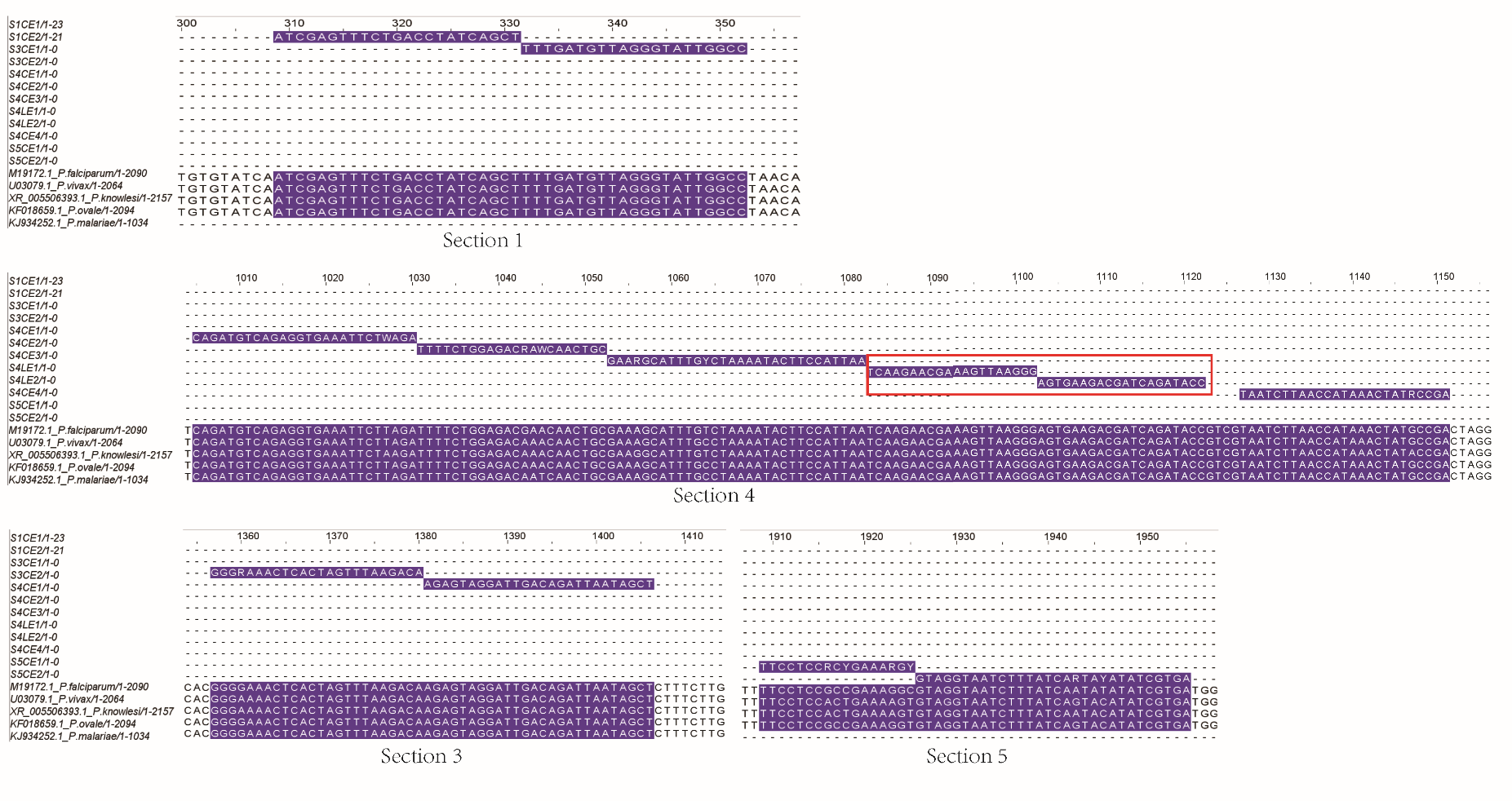


Figure S1 Alignment of capture and ligation probes used for malaria pp-IPA. Sequences with NCBI accession numbers M19172, U03079, XR_005506393, KF018659, and KJ934252 were used as reference sequences for *Plasmodium falciparum*, *Plasmodium vivax*, *Plasmodium knowlesi*, *Plasmodium ovale*, and *Plasmodium malariae*, respectively, to design capture and ligation probes. Sections 1, 3, 4, and 5 represent the selected regions for the design of CP and LP. Sections 1, 3, and 5 each have two capture probes without ligation probes, while section 4 has four capture probes and two ligation probes, with the ligation probes highlighted in red rectangular boxes.


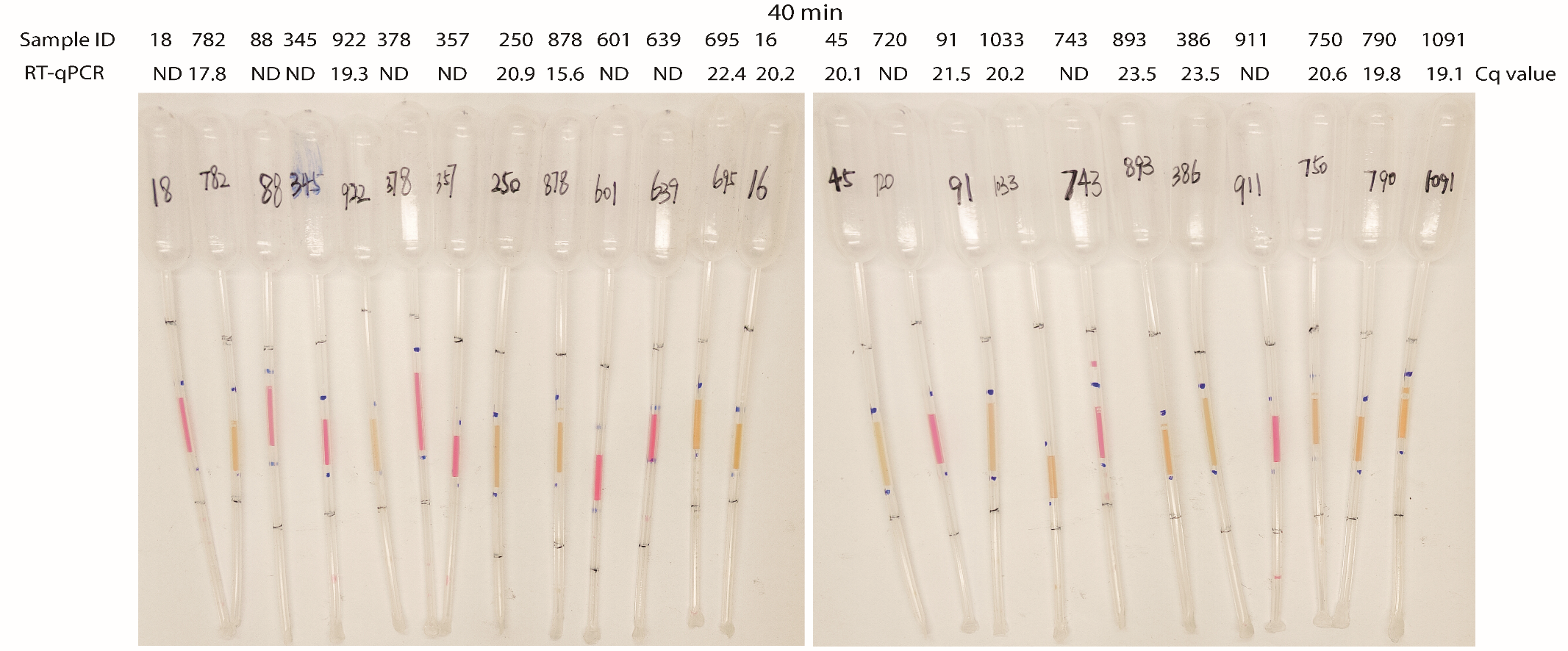


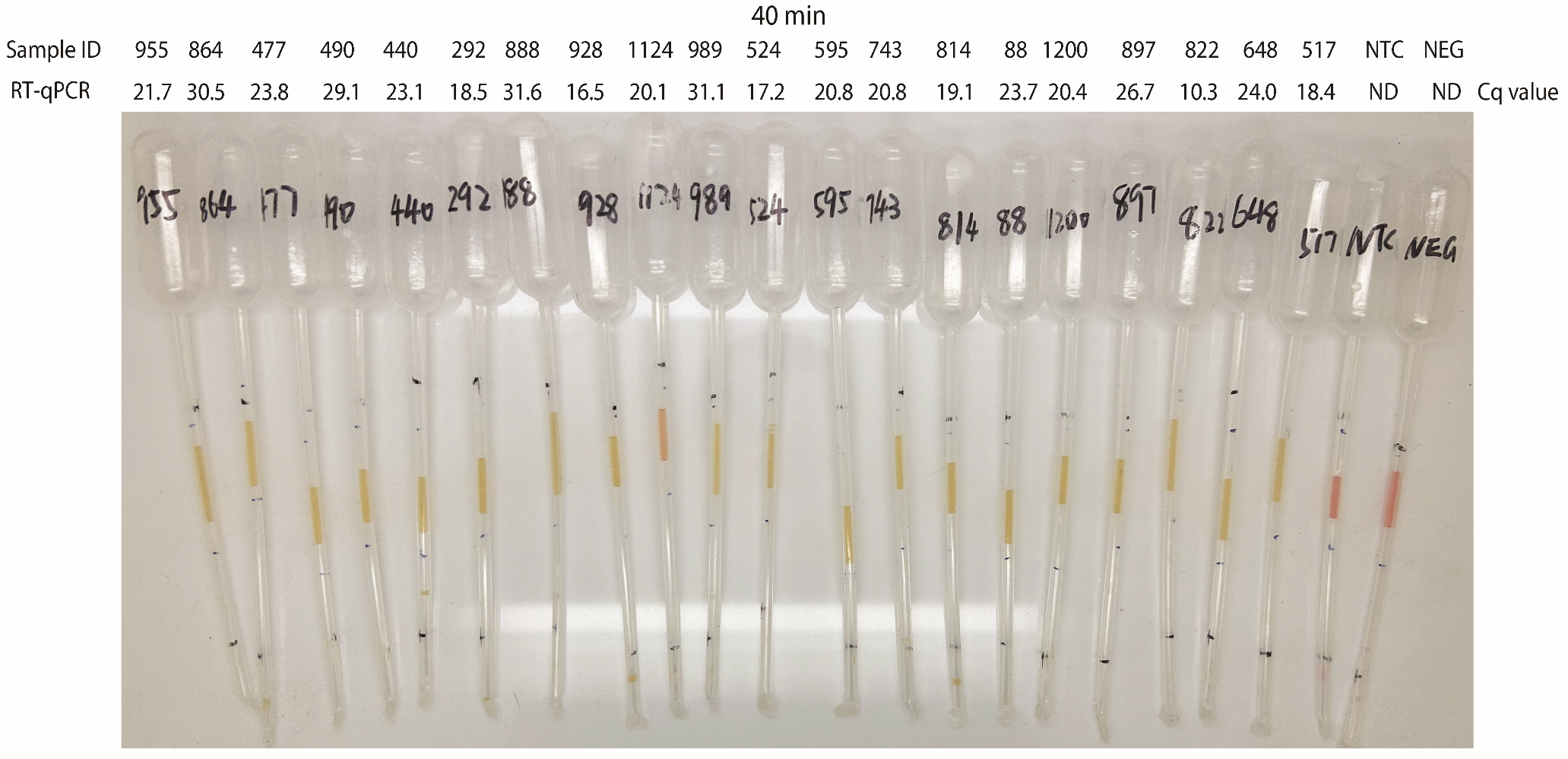


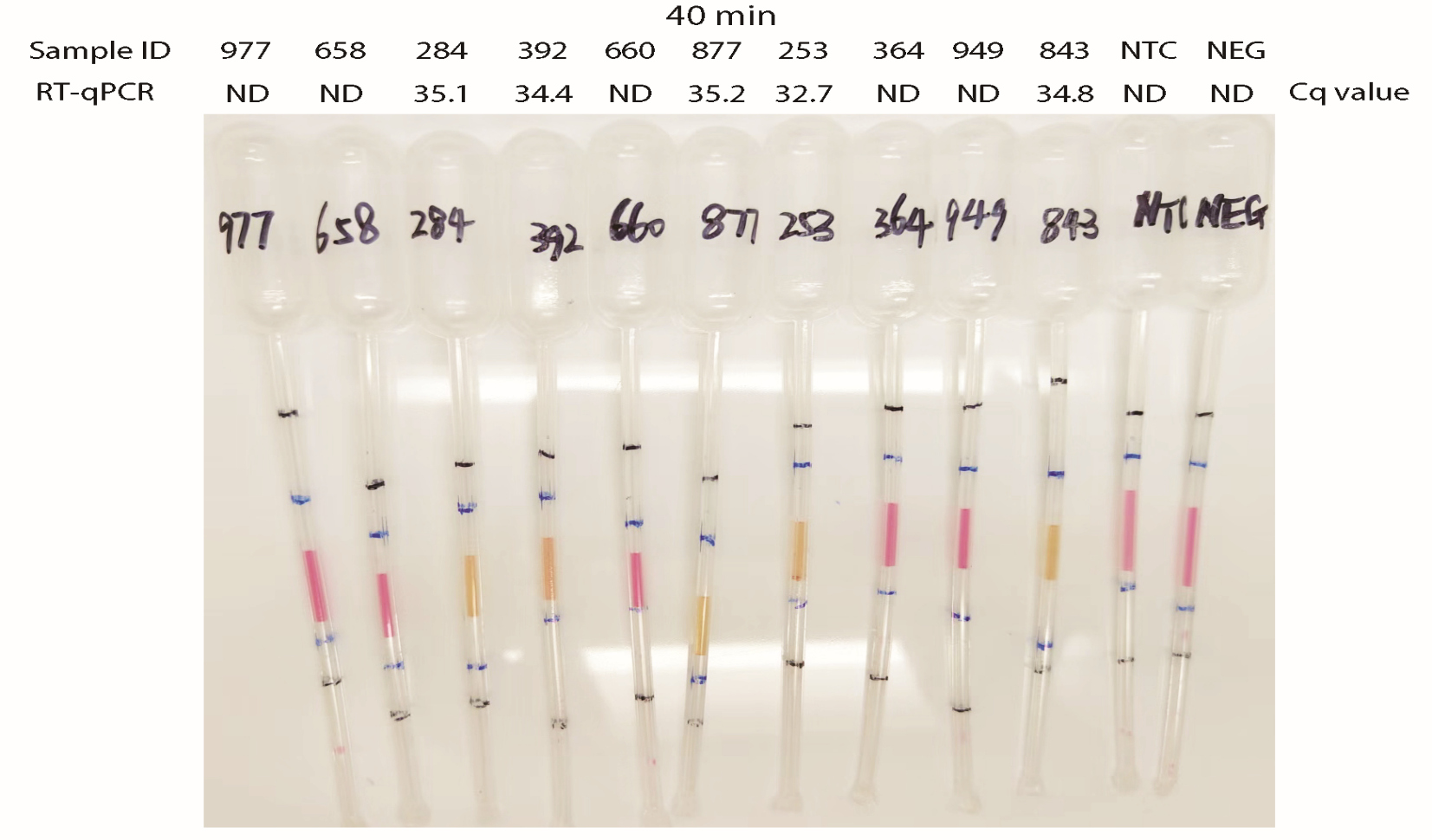


Figure S2 Clinical samples validation by pp-IPA. A total of 54 clinical samples, along with two negative controls, were amplified using pp-IPA. The color change in the reaction mix was observed at three time points: 0 min, 20 min, and 40 min (only the 40 min results are shown; results for 0 min and 20 min are available upon request). The Cq values for each sample, determined by RT-qPCR, are listed below their respective sample ID numbers. ND indicates "not detected," NTC stands for "no template control," and NEG refers to the "negative control."


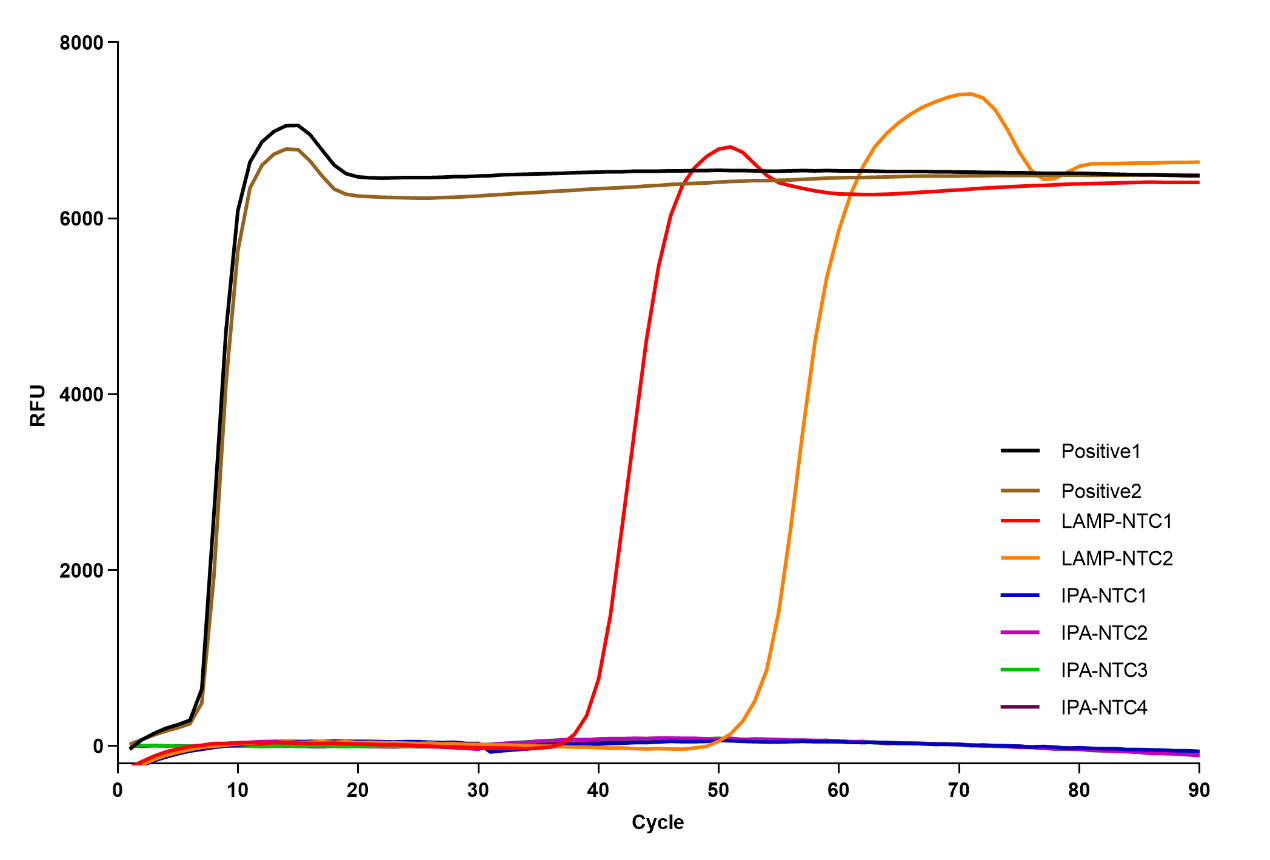


Figure S3 Nonspecific amplification test of IPA. Two positive, two conventional LAMP NTC and 4 IPA NTC were set. Nonspecific amplification occurred at 38 – 50min, no nonspecific amplification occurred for IPA up to 90min.
